# Acquired von Willebrand syndrome and lymphoproliferative disorders: A case report

**DOI:** 10.1002/ccr3.2770

**Published:** 2020-03-09

**Authors:** Christophe Nicol, Leela Raj, Gaëlle Guillerm, Francis Couturaud, Jean‐Richard Eveillard, Brigitte Pan‐Petesch

**Affiliations:** ^1^ Service d'Hématologie Institut de Cancéro‐Hématologie EA3878 (GETBO) CHU Brest Univ Brest Brest France; ^2^ Faculty of Heath Science McMaster University Hamilton ON Canada; ^3^ Département de Médecine Interne et Pneumologie CHU Brest Univ Brest EA 3878, CIC INSERM 1412 Brest France; ^4^ F‐CRIN INNOVTE Brest France

**Keywords:** acquired von Willebrand syndrome, bleeding, daratumumab, smoldering multiple myeloma

## Abstract

Acquired von Willebrand syndrome is a rare bleeding disorder often secondary to an underlying lymphoproliferative disorder. We report a case in whom response of both the acquired von Willebrand syndrome and smoldering multiple myeloma persist 14 months after daratumumab treatment discontinuation.

## Graphical abstract

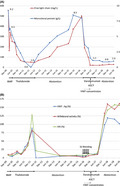

## 1 INTRODUCTION

Acquired von Willebrand Syndrome (AWS) is a rare bleeding disorder associated with multiple conditions including lymphoproliferative malignancies.^1^ An important part of AWS treatment aims to treat the underlying disorder.^2^ However, in patients with asymptomatic lymphoproliferative malignancies, this decision is challenging due to the impact of such therapies on patient quality of life. Furthermore, as the disorder is rare, evidence‐based guidelines regarding when and how to treat underlying conditions are lacking.

We report a case of smoldering multiple myeloma (MM) revealed by AWS, in whom a combination of daratumumab, dexamethasone, and autologous stem cell transplantation led to successful and lasting control of both AWS and MM.

## 2 CASE DESCRIPTION

**Figure 1:**
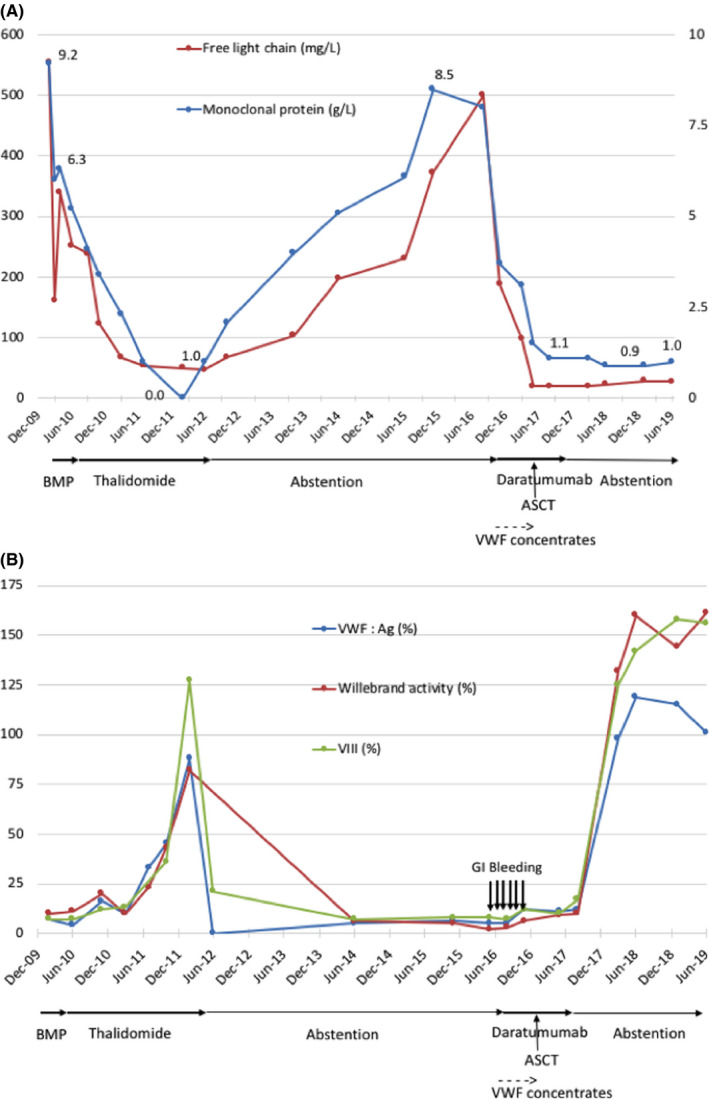
Evolution of multiple myeloma (A) and acquired von Willebrand syndrome (B) from 2010 to 2019. Treatments given are displayed under correct dates. VMP: bortezomib, melphalan, prednisone. ASCT: autologous stem cell transplant. VWF: Rco has been dosed until June 2014, and VWF: Ac has been tested instead from June 2014 until now

Figure 1 displays the evolution of the patient's biological results.

A 60‐year‐old patient with a medical history of tonsillectomy only and neither familial nor personal history of bleeding diathesis presented to our hospital in December 2009 for mild mucocutaneous bleeding. The patient also reported an extended nontraumatic hematoma on his arm several months prior.

Coagulation tests revealed normal prothrombin time, prolonged activated partial thromboplastin time (ratio = 1.56), normal fibrinogen levels, prolonged platelet occlusion time with (both inductors [epinephrin, adenosine diphosphate] > 300 seconds), low levels of von Willebrand factor (VWF) antigen (VWF: Ag = 7%; NV = l65%‐200%; STAGO STA Latest VWF AG), factor VIII (FVIII = 7%; NV = 65%‐200%; STAGO STA CK Prest 5, Stago Deficient VIII), and VWF ristocetin cofactor (VWF: Rco = 10%; NV = 65‐200). High molecular weight VWF multimers were not tested. Blood group was A Rhesus positive. Hemoglobin, white cell count, platelets, TSH, creatinine, albumin, and calcium values were normal.

A monoclonal protein spike of IgG Kappa type at 9.2 g/L was detected in the serum, with elevated free kappa chains (500 mg/dL), an elevated serum free light ratio (30), and no urinary protein abnormality. Bone marrow aspiration revealed abnormal plasmocytes (>10% hyperdiploid on cytogenetics). No bone lytic lesions were found on X‐ray.

A diagnosis of AWS secondary to IgG Kappa smoldering MM was made. Initial treatment through desmopressin and intravenous immunoglobin (IVIg) was started in February 2010 but proved ineffective. A decision to treat the MM due to bleeding symptoms was made.

A weekly bortezomib, melphalan, and prednisone (BMP) treatment was started in March 2010. Gum oozing and bleeding ceased after one cycle. However, lack of improvement in coagulation screening tests after 3 cycles led to a decision to opt for thalidomide treatment instead.

In February 2012, serum monoclonal protein levels remained detectable but unquantifiable while coagulation tests revealed normal. However, debilitating peripherical neuropathy led to discontinuation of thalidomide therapy.

Four months later, gum bleeding resumed. A drop in VWF: Ag and a mild increase of monoclonal protein were simultaneously observed. Patient preference and mildness of bleeding led to therapeutic abstention.

In May 2013, administration of VWF concentrate, having shown a favorable response in October, 2011, was ineffective as demonstrated by the unusually rapid drop in VWF: Ag after injection, suggesting a half‐life of <1 hour compared to the usual 8‐12 hour half‐life.

Between April and October 2016, six gastrointestinal bleeding episodes occurred suggesting a gastric ulcer or cecal lesion, and led to severe anemia.

Although the MM remained asymptomatic, our team decided to resume treatment through a novel combination of daratumumab, dexamethasone, and autologous stem cell transplantation due to bleeding. This decision was based on the recommendations of French national experts who considered the patient at high risk of recurrent and life‐threatening bleeding complications and suggested that this latter therapeutic combination could simultaneously eradicate the tumoral clone while exerting lasting control over the AWS.

Prophylactic treatment of bleeding consisted of tranexamic acid, somatuline, and human plasmatic VWF concentrate started at 80 IU/kg thrice a day. VWF concentrate dose was progressively reduced until autologous stem cell transplantation conditioning regimen by melphalan 200 mg/m^2^ was attained, with temporary increase during aplasia and discontinuation upon achievement of platelet count >50 g/L. A total of 24 infusions of daratumumab were administered. Twenty‐two months after the last infusion, monoclonal protein remains stable at 0.9 g/L and coagulation tests remain normal. No bleeding occurred since prophylactic VWF concentrate discontinuation.

## 3 DISCUSSION

Our patient, who received a double diagnosis of smoldering MM and AWS after presenting with hemorrhagic symptoms, illustrates the challenges in the management of lymphoproliferative malignancy‐associated AWS due to the lack of standardized treatment guidelines. Currently, such patients are treated according to two broad recommendations: (a) managing bleeding symptoms and (b) treating the underlying condition by targeting the AWS mechanism.^2^

Primary hemorrhage management is usually done through tranexamic acid. Longer‐term hemorrhage prevention generally involves desmopressin, VWF‐containing concentrates or plasmapheresis.^3^, ^4^, ^5^, ^6^ Recently, IVIg has also demonstrated promising results as maintenance therapy.^7^, ^8^ In our patient, both desmopressin and IVIg were ineffective, and successful bleeding management was achieved using a combination of tranexamic acid, somatuline and human plasmatic VWF concentrate.

Decision to treat the underlying asymptomatic lymphoproliferative malignancy in an AWS patient poses a complex dilemma. While it is believed that there exists a physiological relationship between both conditions, further investigation on these conjectures remains to be done. Additionally, responses of each condition to these treatments have shown discordances and important patient dependence. Thus, for clinicians, the decision to subject patients to the highly side effect‐associated treatments commonly used in otherwise asymptomatic lymphoproliferative malignancies is conflicting.

In our patient, decision to begin treatment for smoldering MM was based on the relative importance of bleeding symptoms. Initial treatment through BMP led to clinical but not biological improvement of AWS and partial amendment of biological manifestation of MM, illustrating the discordant responses of both disorders.

Subsequent use of thalidomide in our patient led to both clinical and biological effectiveness in AWS and smoldering MM. However, effects were not maintained as revealed by the recurrence of AWS manifestation as acute gastrointestinal hemorrhage, thus prompting our decision to opt for an innovative combination of daratumumab, dexamethasone, and autologous stem cell transplantation as third‐line therapy. Treatment results exceeded our expectations, with biological and symptomatic treatment effects lasting nearly 2 years past final administration.

To our knowledge, ours is an unprecedented use of daratumumab in a patient with MM‐associated AWS.^9^, ^10^, ^11^, ^12^, ^14^, ^15^, ^16^ Our results have potentially important implications in guiding the treatment of patients with asymptomatic plasma cell disorder‐associated AWS.

Daratumumab has recently been approved in the treatment of MM and may offer an attractive solution to the clinical dilemma in patients such as ours.^13^ This first class human IgG1 monoclonal CD38‐binding antibody has demonstrated impressive effectiveness on patients having been refractory to immunomodulators and proteasome inhibitors.^17^ This indicates the likelihood of a novel mechanism of action of daratumumab and may explain the concordant symptomatic and biological responses of both the MM and AWS to this treatment by our patient in contrast to those observed with the previous lines of treatment.^17^ Thus, these findings lend support to our hypothesis that daratumumab, unlike other commonly used MM treatments, may be able to simultaneously target both the MM and AWS with more consistent and predictable results. Furthermore, daratumumab's favorable safety profile and lack of overlapping toxicity when administered in combination with other antimyeloma agents make this drug particularly attractive.^13^ Taken together, the likelihood of a novel mechanism simultaneous targeting the MM and AWS and the favorable toxicity profile of daratumumab provide positive prospects to its wider adoption in complex patients presenting with asymptomatic plasma cell disorder‐associated AWS.

While our results are promising, we advise caution in their widespread application as our findings are based on a single case and patient‐dependent factors have therefore undeniably played a role in our results. Such favorable responses in all MM‐associated AWS patients subjected to treatment through daratumumab cannot be guaranteed. Nonetheless, findings of our report strongly suggest consideration of daratumumab for clinicians faced with MM‐associated AWS patients having proven refractory to previous lines of therapy and we suggest further inquiry into the possibility of employing daratumumab as first‐line therapy in this patient profile.

## 4 CONCLUSION

Our case illustrates the challenges facing clinicians in administering lymphoproliferative malignancy therapy in lymphoproliferative malignancy‐associated AWS on the sole indication of bleeding, due to the impact these therapies on patient quality of life and lack of specific guidelines. In our patient, a novel combination of the newly approved daratumumab and dexamethasone, concomitantly with autologous stem cell transplantation exceeded expected results. Our findings suggest the need for further investigation on the role and effectiveness of daratumumab‐based therapies in lymphoproliferative malignancy‐associated AWS.

## CONFLICT OF INTEREST

All authors have completed and submitted the ICMJE Form for Disclosure of Potential Conflicts of Interest. Ms Raj declares she has no conflict of interest related to this research. Dr Nicol declares he has no conflict of interest related to this research. Dr Guillerm declares she has no conflict of interest related to this research. Dr Couturaud reports having received research grant support from Pfizer and fees for board memberships or symposia from Bayer, Bristol‐Myers Squibb/Pfizer and Astra Zeneca and having received travel support from Bayer, Bristol‐Myers Squibb/Pfizer, Daiichi Sankyo, Boehringer Ingelheim, Leo Pharma, Intermune and Actelion. Dr Eveillard declares he has no conflict of interest related to this research. Dr Pan‐Petesch reports having received fees for board membership or symposia from Takeda, Roche, Sobi et Novartis, CSL‐Berhing and having received travel support from Octapharma,, Novonordisk, Sobi, Takeda Shire, Novartis, Amgen, Biomarin, LFB, CSL‐Berhing.

## AUTHOR CONTRIBUTIONS

BPP: had full access to all of the data in the study and takes responsibility for the integrity of the data and the accuracy of the data analysis. CN, BPP: conceived and designed the study. CN, GG, JRE, BPP: acquired data. CN, LR, FC, BPP: drafted the manuscript. GG, FC, JRE, BPP: provided administrative, technical, or material support. BPP: supervised study. All the authors analyzed and interpreted the data; critically revised the manuscript for important intellectual content; and involved in the final approval of the manuscript.
